# Circadian control of bile acid synthesis by a KLF15-*Fgf15* axis

**DOI:** 10.1038/ncomms8231

**Published:** 2015-06-04

**Authors:** Sean (Shuxin) Han, Rongli Zhang, Rajan Jain, Hong Shi, Lilei Zhang, Guangjin Zhou, Panjamaporn Sangwung, Derin Tugal, G. Brandon Atkins, Domenick A. Prosdocimo, Yuan Lu, Xiaonan Han, Patrick Tso, Xudong Liao, Jonathan A. Epstein, Mukesh K. Jain

**Affiliations:** 1 Department of Medicine, Case Cardiovascular Research Institute, Case Western Reserve University, and Harrington Heart and Vascular Institute, University Hospitals Case Medical Center, Cleveland, Ohio 44106, USA; 2 Department of Cell and Developmental Biology, Perelman School of Medicine at the University of Pennsylvania, Philadelphia, Pennsylvania 19104, USA; 3 Penn Cardiovascular Institute, Perelman School of Medicine at the University of Pennsylvania, Philadelphia, Pennsylvania 19104, USA; 4 Institute of Regenerative Medicine, Perelman School of Medicine at the University of Pennsylvania, Philadelphia, Pennsylvania 19104, USA; 5 Division of Gastroenterology, Hepatology and Nutrition, Cincinnati Children’s Hospital Medical Center, Cincinnati, Ohio 45229, USA; 6 Mouse Metabolic Phenotype Center, Department of Pathology and Laboratory Medicine, Metabolic Diseases Institute and the University of Cincinnati College of Medicine, Cincinnati, Ohio 45229, USA

## Abstract

Circadian control of nutrient availability is critical to efficiently meet the energetic demands of an organism. Production of bile acids (BAs), which facilitate digestion and absorption of nutrients, is a major regulator of this process. Here we identify a KLF15-*Fgf15* signalling axis that regulates circadian BA production. Systemic *Klf15* deficiency disrupted circadian expression of key BA synthetic enzymes, tissue BA levels and triglyceride/cholesterol absorption. Studies in liver-specific *Klf15*-knockout mice suggested a non-hepatic basis for regulation of BA production. Ileal *Fgf15* is a potent inhibitor of BA synthesis. Using a combination of biochemical, molecular and functional assays (including ileectomy and bile duct catheterization), we identify KLF15 as the first endogenous negative regulator of circadian *Fgf15* expression. Elucidation of this novel pathway controlling circadian BA production has important implications for physiologic control of nutrient availability and metabolic homeostasis.

## Abstract (web summary)

Bile acids are important for the absorption of nutrients. Here the authors provide a molecular explanation for the oscillatory release of bile acids, showing that diurnal expression of the transcription factor KLF15 regulates FGF15 secretion from enterocytes, which then inhibits bile acid synthesis in the liver.

## Graphical abstract

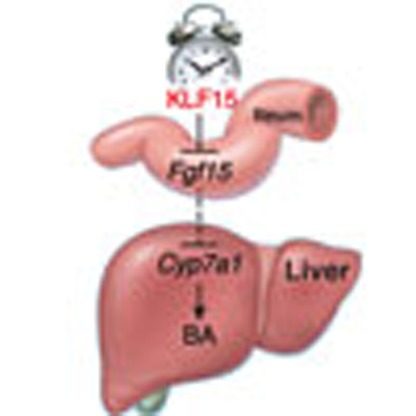

Bile acids (BAs) are derived from enzymatic oxidation of cholesterol and function as detergents that facilitate digestion and absorption of nutrients^1^,^2^. In addition, there is growing appreciation that BA can function as hormones to regulate systemic metabolic homeostasis^3^. Previous studies have demonstrated that BA production exhibits a distinct daily rhythm^4^,^5^,^6^,^7^ but our understanding of endogenous mechanisms that regulate this process are incompletely understood.

Primary BA are synthesized in the liver, stored temporarily in the gallbladder (GB), secreted into the intestine on food ingestion (to facilitate absorption of dietary lipids and fat-soluble vitamins) and then reabsorbed in the distal ileum. In addition, non-hepatic sources of BAs such as microbiota can affect BA composition and pools^8^. With respect to hepatic BA production, the major and rate-limiting enzyme in BA production is cholesterol 7α-hydroxylase (*Cyp7a1)*, with minor contributions from additional enzymes such as 25-hydroxycholesterol 7α-hydroxylase (*Cyp7b1*)^2^. Importantly, reabsorption of BAs in the distal ileum is critical as part of a negative feedback loop that inhibits BA synthesis in the liver by repressing *Cyp7a1*. Studies over the past decade have revealed that feedback inhibition of BA synthesis involves ileal farnesoid X receptor (FXR/NR1H4)-induced fibroblast growth factor 15 (*Fgf15*)^9^ and hepatic FXR-induced atypical nuclear receptor small heterodimer partner (*Shp/Nr0b2*)^10^. These studies indicate that expression of *Fgf15*, which occurs principally in enterocytes of the ileum^11^,^12^, is induced by BA-mediated activation of the nuclear receptor FXR in enterocytes. FGF15 is subsequently secreted into the circulation where it is transported to the liver and binds its cognate receptor fibroblast growth factor receptor 4/FGFR4 and co-receptor βKlotho on hepatocytes^13^ and activates a signalling pathway that culminates in repression of *Cyp7a1*.

Efforts to understand the circadian basis for BA synthesis have focused on CYP7A1 as this key enzyme exhibits robust circadian expression and activity during a day–night cycle. Several circadian transcription factors have been identified as direct transcriptional regulators of *Cyp7a1* messenger RNA (mRNA) expression in hepatocytes^4^,^5^. Although several recent reports have documented that *Fgf15/19* levels exhibit diurnal variation^14^,^15^, the molecular basis and functional importance in regulating circadian BA production is unknown.

## Results

### KLF15 regulates BA synthesis

**Figure 1:**
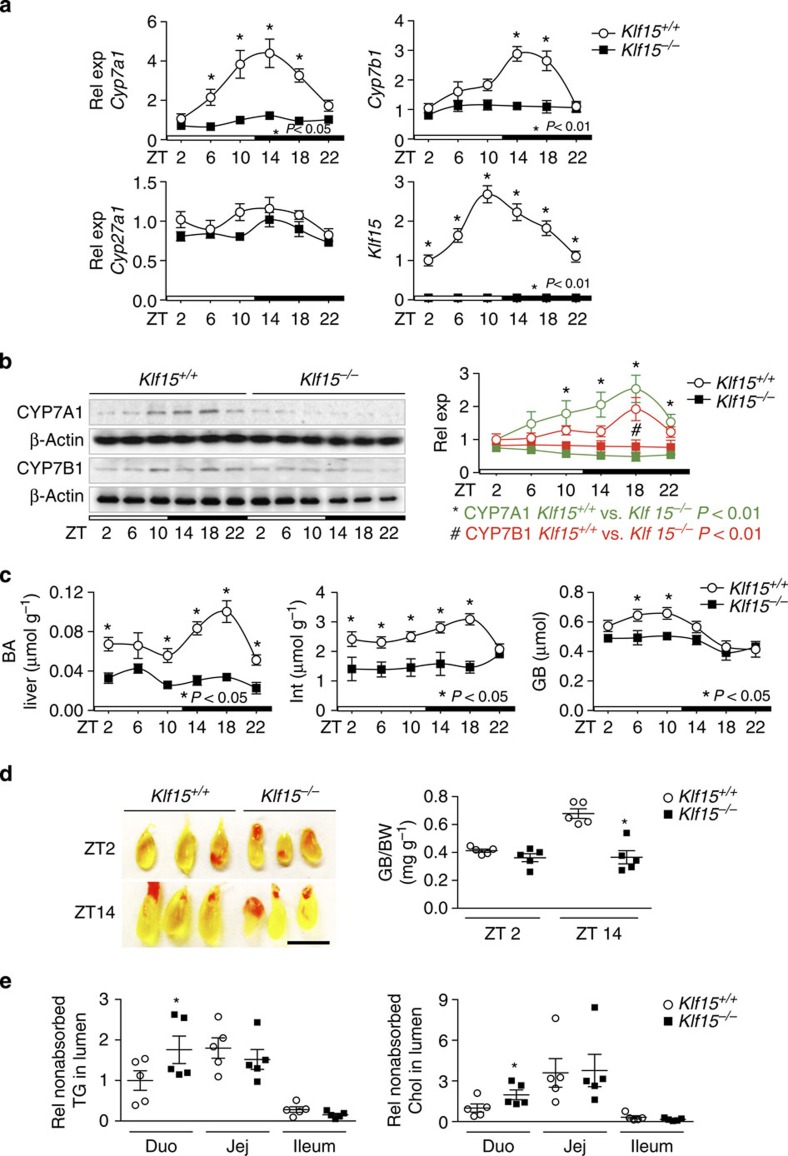
*Klf15* deficiency attenuates circadian bile acid (BA) synthesis and lipid absorption. (**a**) The circadian mRNA relative expression (Rel Exp) of BA synthetic enzymes *Cyp7a1*, *Cyp7b1* and *Cyp27a1* as well as *Klf15* in wild-type (*Klf15*^*+/+*^) and knockout (*Klf15*^*−/−*^) mouse livers (five per time point). *Cyp7a1*, *Cyp7b1* and *Klf15* mRNA expressions exhibit endogenous circadian rhythms in *Klf15*^*+/+*^ mouse livers (*P*<0.01) but the rhythms in *Klf15*^*−/−*^ livers were lost with reduced expression at indicated time points. ZT, zeitgeber time (h). (**b**) Immunoblot (left) and quantification (right) of CYP7A1 and CYP7B1 protein expressions in mouse livers (representative of three experiments). The Rel Exps of CYP7A1 and CYP7B1 proteins (right) exhibit diurnal rhythm in *Klf15*^*+/+*^ mouse livers (*P*<0.05) but the rhythms in *Klf15*^*−/−*^ livers were lost with reduced expression at indicated time points. (**c**) BA amount in liver, intestine (Int) and gallbladder (GB) monitored in a circadian fashion (*n*=5). Liver and intestine BAs amount were normalized to tissue mass. The tissue BA fractions exhibit diurnal rhythm in *Klf15*^*+/+*^ mice (*P*<0.01) but not in *Klf15*^*−/−*^ mice with reduced BA amount at indicated time points. (**d**) GB sizes and weights normalized to body weights at ZT2 or ZT14 (*n*=5). Scale bar, 5 mm. (**e**) Triglyceride (TG) and cholesterol (Chol) absorption in small intestine. Relative amount of non-absorbed TG and Chol in three small intestinal segments: duodenum (Duo), jejunum (Jej) and ileum (*n*=5). Data represent mean±s.e.m. Statistical significance of circadian rhythm for each genotype was analysed using analysis of variance (one way within genotype; two way between genotypes) followed by Bonferroni post-test. Statistical significance between the two individual groups was assessed using Student’s *t*-test. Normal distribution was assumed for all experimental groups. * and # indicate *P*<0.05 or 0.01, compared with *Klf15*^*+/+*^ mice at indicated time points or relative (Rel) non-absorbed TG or cholesterol detected in *Klf15*^*+/+*^ mouse duodenum.

Recent work has identified the transcription factor Kruppel-like factor 15 (KLF15), as critical for nutrient flux and utilization in the context of daily feed–fast cycles^16^. Unbiased transcriptome analysis of mouse livers from wild-type (*Klf15*^*+/+*^) and *Klf15* systemic knockout mice (*Klf15*^*−/−*^) revealed reduced expression of *Cyp7a1* and *Cyp7b1* (ref. 17). To confirm these findings, liver tissues were harvested from control and systemic *Klf15*^*−/−*^ mice at 4-h intervals across a 24-h cycle (ZT0: 06:00, lights on; ZT12: 18:00, lights off). As expected, *Klf15* and several BA synthetic enzymes exhibited an oscillatory expression pattern (Fig. 1a,b). Importantly, the oscillation of *Cyp7a1* and *Cyp7b1* mRNA and protein were attenuated in *Klf15*^*−/−*^ livers with minimal effect on sterol 27-hydroxylase (*Cyp27a1*; Fig.1a). Consistent with this reduction in *Cyp7a1*, tissue BA levels as well as GB weight were reduced in *Klf15*^*−/−*^ mice (Fig. 1c,d). No effect was found on mRNA expression of key factors known to regulate *Cyp7a1* (Supplementary Fig. 1). As BAs are critical for lipid absorption, we assessed the effect of *Klf15* deficiency on triglyceride (TG) and cholesterol absorption. Labelled TG and cholesterol were infused into the gut and luminal amounts assessed 6 h after infusion. *Klf15*^*−/−*^ mice exhibited higher levels of luminal lipids (both TG and cholesterol) in the duodenum, one of the main regions of the gastrointestinal tract involved in absorbing lipid-soluble nutrients (Fig. 1e). The presence of higher amount of TG or cholesterol in the duodenal lumen indicated reduced absorption, a finding consistent with the observation that BAs are decreased in the *Klf15*^*−/−*^ animals (Fig. 1c). Collectively, these findings identify KLF15 as an essential regulator for circadian expression of key BA synthetic enzymes, BA pools and fat absorption.

As both *Klf15* and *Cyp7a1* are robustly expressed in the liver, we hypothesized that hepatic KLF15 likely regulated *Cyp7a1* at the transcriptional level. However, co-transfection studies failed to show any effect of KLF15 on *Cyp7a1*-*luciferase* reporter activity at baseline or in combination with several known positive regulators of *Cyp7a1* (Supplementary Fig. 2a–d). Further, viral overexpression or knockdown of *Klf15* in hepatocytes had only a modest effect on *Cyp7a1* expression (Supplementary Fig. 2e,f). To definitively determine whether hepatic KLF15 regulates *Cyp7a1* and BA synthesis, we generated liver-specific *Klf15*-knockout mice (Li-KO; *Klf15*^*f/f*^*; Albumin-Cre* (*Alb-Cre*)). Efficient deletion of *Klf15* was confirmed at both the mRNA and protein levels (Supplementary Fig. 3a,b). Consistent with our *in vitro* studies, Li-KO mice demonstrated only a minimal alteration in *Cyp7a1* mRNA expression and BA pools in the tissues (Supplementary Fig. 3c,d). By contrast, oscillation of the minor BA regulatory enzyme *Cyp7b1* was modestly reduced (Supplementary Fig. 3c, right panel). These findings suggest a non-hepatic basis for KLF15 regulation of *Cyp7a1* expression and BA synthesis.

### Ileal KLF15 inhibits *Fgf15*

**Figure 2:**
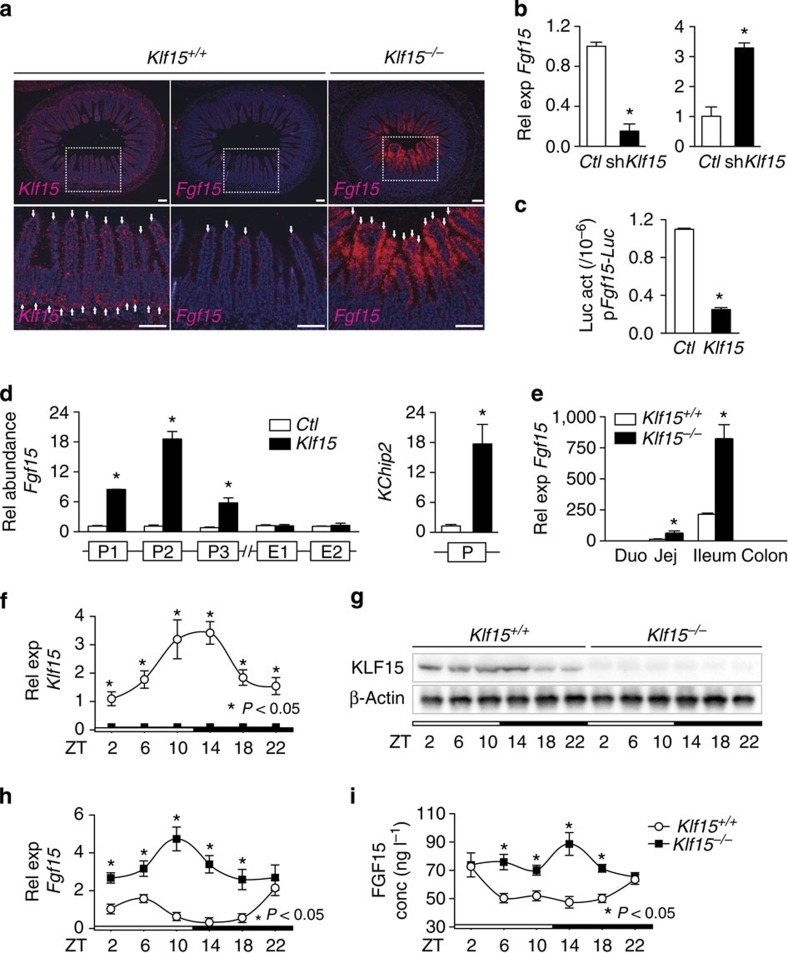
Ileal KLF15 regulates circadian FGF15 production to control BA synthesis. (**a**) *In situ* hybridization analysis of *Klf15* and *Fgf15* expression in *Klf15*^*+/+*^ and *Klf15*^*−/−*^ mouse ileum. White arrows indicate *Klf15* or *Fgf15* mRNA signal. (**b**) Effects of adenoviral overexpression and shRNA knockdown of *Klf15* on *Fgf15* mRNA expression in primary mouse small intestinal epithelial cells. Primary small intestinal epithelial cells were infected with control virus (*Ctl*), ad*Klf15*, or sh*Klf15* as indicated. (**c**) *Fgf15-Luc* reporter assay in Caco2 cells following heterologous overexpression of *Flag-Klf15* (*Klf15*) or *pc-DNA3.1* (control, *Ctl*). (**d**) ChIP analysis of KLF15 (*Flag-Klf15*) binding to designate regions of the *Fgf15* and the Kv channel-interacting protein 2 (*KChip2*) promoters in the mouse colon cell line CT26. The relative binding abundance (Rel abundance) was normalized to IgG. (**e**) *Fgf15* mRNA expression in *Klf15*^*+/+*^ versus *Klf15*^*−/−*^ mouse intestine segments: Duo, Jej, ileum and colon (*n*=8). (**f**) Circadian rhythm of *Klf15* mRNA expression in *Klf15*^*+/+*^ mouse ileum (*P*<0.01) was abolished in *Klf15*^*−/−*^ mouse ileum (*n*=5 per time point). (**g**) Immunoblot of circadian KLF15 protein expression in the ileum (representative of three experiments). Ileal *Fgf15* mRNA expression (**h**) and blood FGF15 protein concentrations (Conc) (**i**) exhibit circadian variations in *Klf15*^*+/+*^ mice (*P*<0.01) but the rhythms in *Klf15*^*−/−*^ mice were lost with increased expression at indicated time points (*n*=5). Data represent mean±s.e.m. Statistical significance of circadian rhythm for each genotype was analysed using analysis of variance followed by Bonferroni post-test. Statistical significance between the two individual groups was assessed using Student’s *t-*test. * indicates *P*<0.05, compared with control (*Ctl*), or *Klf15*^*+/+*^ mice. Scale bars, 100 μm (**a**).

An essential, non-hepatic mechanism regulating *Cyp7a1* expression and BA synthesis involves ileum-derived FGF15. Previous studies show that *Fgf15* is expressed in the epithelial cells of the ileum^9^ but the cellular expression of *Klf15* in the ileum is unknown. *In situ* hybridization (ISH) studies show that both *Fgf15* and *Klf15* mRNA are expressed in the epithelial cells of ileal villi (Fig. 2a; Supplementary Fig. 4a). Overexpression of *Klf15* in primary mouse small intestinal epithelial cells potently repressed, while knockdown induced, *Fgf15* expression at baseline or following induction of *Fgf15* by FXR agonist GW4064 (Fig. 2b; Supplementary Fig. 4b–d). Gene reporter assays revealed that KLF15 repressed the *Fgf15* promoter (Fig. 2c) and chromatin immunoprecipitation (ChIP) analyses confirmed significant KLF15 occupancy at multiple consensus binding sites (CACCC or G/C-rich elements) within the endogenous *Fgf15* promoter (Fig. 2d; the *KChip2* promoter serves as positive control^18^). In addition, *Fgf15* was strongly induced in the *Klf15*^*−/−*^ ileum (and modestly in jejunum) as assessed by quantative PCR (qPCR) analysis (Fig. 2e) and confirmed by ISH (Fig. 2a, far right panel). Finally, given that KLF15 is a circadian factor, we assessed *Klf15* and *Fgf15* expression across a 24-h cycle. As shown in Fig. 2f–i, *Klf15* and *Fgf15* mRNA and protein expressions (by western blot or plasma enzyme-linked immunosorbent assay (ELISA)) both oscillated in ileal tissues and blood. Importantly, in the absence of *Klf15*, ileal *Fgf15* mRNA expression and serum FGF15 levels were increased at multiple time points across a 24-h cycle. Collectively, these data identify KLF15 as a direct negative regulator of *Fgf15*.

### Ileectomy restores BA synthesis in *Klf15* ^*−/−*^ mice

**Figure 3:**
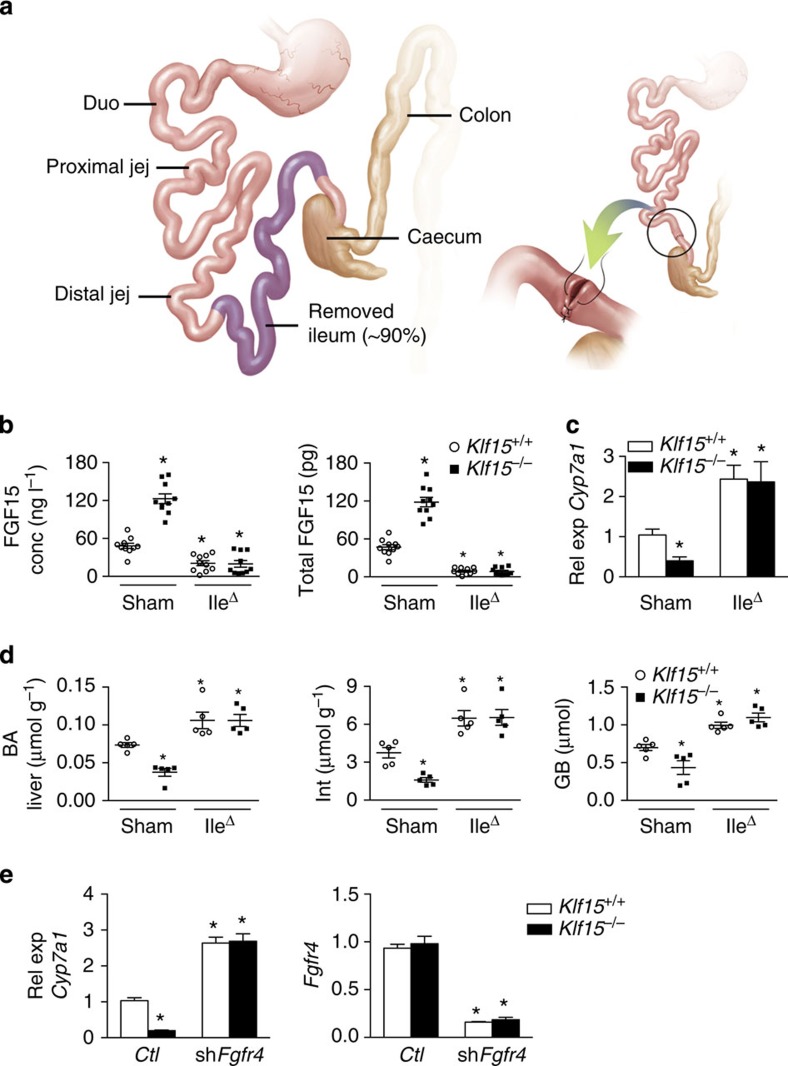
Ileectomy and knockdown of hepatic *Fgfr4* restore *Cyp7a1* expression and BA synthesis in *Klf15*^*−/−*^ mice. (**a**) Schematic picture before and after ileectomy (Ile^Δ^). Approximately 90% of the ileum was resected. (**b**) FGF15 protein concentration (left) and total amount (right) in blood from *Klf15*^*+/+*^ and *Klf15*^*−/−*^ mice at ZT14 after sham or Ile^Δ^ surgery (*n*=10). (**c**) The mRNA levels of hepatic *Cyp7a1* in *Klf15*^*+/+*^ and *Klf15*^*−/−*^ mice at ZT14 after sham or Ile^Δ^ (*n*=5). (**d**) BA amount in liver, intestine (Int) and GB in *Klf15*^*+/+*^ versus *Klf15*^*−/−*^, sham versus Ile^Δ^ mice (*n*=5). (**e**) mRNA expression of the liver *Cyp7a1* and *Fgfr4* in *Klf15*^*+/+*^ and *Klf15*^*−/−*^ mice infected with adenoviral shRNA control (*Ctl*) or sh*Fgfr4* (*n*=5). Statistical significance between the two individual groups was assessed using Student’s *t*-test. Normal distribution was assumed. * indicates *P*<0.05, compared with *Klf15*^*+/+*^ mice with sham surgery or infected with *Ctl*.

The above data are consistent with a model in which ileal KLF15 controls *Cyp7a1* expression in the liver by regulating ileal *Fgf15*. Resection of ileum restored plasma FGF15 levels, *Cyp7a1* mRNA expression and BA amounts in *Klf15*^*−/−*^ mice to levels comparable to *Klf15*^*+/+*^ mice (Fig. 3a–d). Similar results were found after both ileal and jejunal resection (Supplementary Fig.5a,b). As FGF15 binding to its receptor FGFR4 is critical for inhibition of *Cyp7a1* and BA production, we next sought to determine whether FGFR4 was required for KLF15-mediated downregulation of *Fgf15*. Tail-vein injection of adenoviral *Fgfr4* short hairpin RNA (shRNA) efficiently reduced mRNA expression by ∼90% (Fig. 3e). Critically, the reduced expression of *Cyp7a1* in *Klf15*^*−/−*^ mice was completely abrogated to levels comparable to those observed in *Klf15*^*+/+*^ mice following *Fgfr4* depletion. Collectively, the results of Figs 2 and 3 suggest that KLF15-dependent regulation of ileal *Fgf15* is critical for *Cyp7a1* expression and BA synthesis in the liver.

### KLF15 regulates FGF15 and BA synthesis in a BA-independent manner

**Figure 4:**
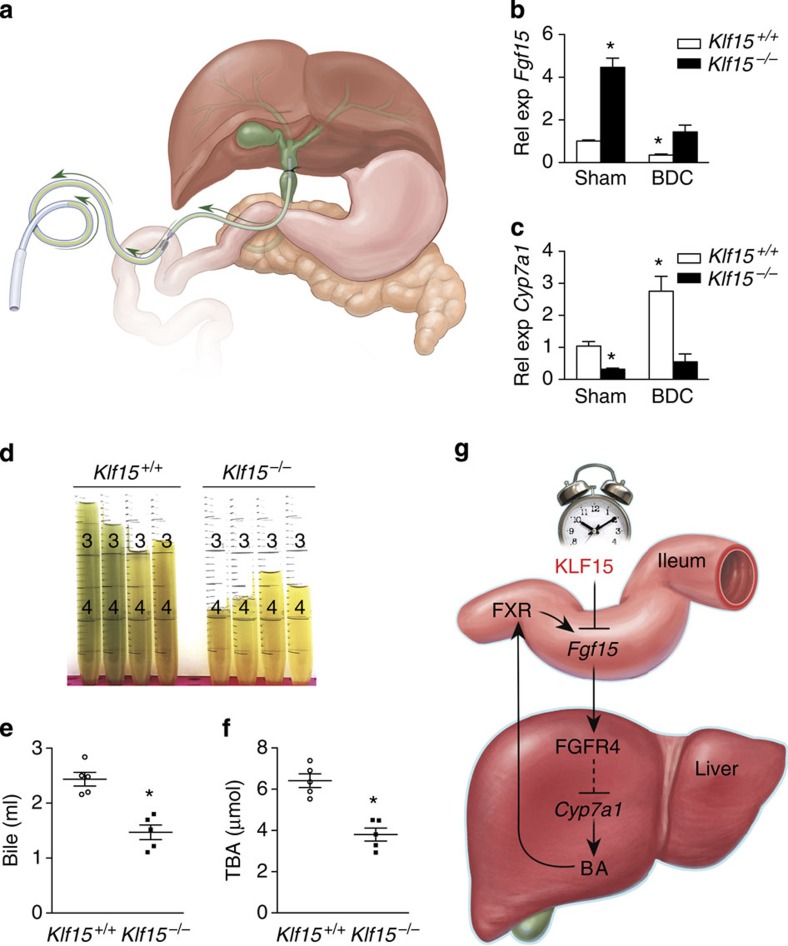
KLF15 inhibits *Fgf15* and induces BA synthesis in a BA-independent manner. (**a**) Schematic representation of bile duct catheterization (BDC). mRNA expression of intestinal *Fgf15* **(b)** and liver *Cyp7a1* **(c)** in *Klf15*^*+/+*^ and *Klf15*^*−/−*^ mice at ZT14 after sham or BDC surgery (*n*=5). (**d**) Bile collected from *Klf15*^*+/+*^ and *Klf15*^*−/−*^ mice 24 h after BDC surgery (one mouse bile/tube). (**e**) Bile total volume in *Klf15*^*+/+*^ and *Klf15*^*−/−*^ mice 24 h after BDC surgery. (**f**) Total BA (TBA) amount in collected *Klf15*^*+/+*^ and *Klf15*^*−/−*^ mouse bile (*n*=5 per group). (**g**) Schematic model of KLF15 regulation of mouse *Cyp7a1* gene expression and BA synthesis. Statistical significance between the two individual groups was assessed using Student’s *t*-test. Normal distribution was assumed. * indicates *P*<0.05, compared with sham or *Klf15*^*+/+*^ mice.

Recent literature suggests that BA-dependent activation of FXR in epithelial cells of the distal ileum leads to *Fgf15* production. However, our mechanistic data raises the possibility that KLF15 may directly regulate *Fgf15*. If this hypothesis is correct, then the effect of *Klf15* deficiency on *Fgf15* and *Cyp7a1* expression and BA production should be preserved in the absence of BA. To address this hypothesis, we employed a bile duct catheterization (BDC) model in mice that captures BAs before they can enter the small bowel (Fig. 4a; Supplementary Fig. 6a). This approach also allows for quantification of BA production while avoiding BA-induced hepatic toxicity that may occur with ligation of the bile duct. Importantly, the oscillation of ileal *Fgf15*, ileal *Klf15* and hepatic *Cyp7a1* at ZT2 and ZT14 was maintained after BDC (Supplementary Fig. 6b,c). Further, the expected effect of *Klf15* deficiency on *Fgf15*/*Cyp7a1* expression was present after sham surgery (Fig. 4b,c). Importantly, following BDC, the expression of ileal *Fgf15* in *Klf15*^*−/−*^ animals remained significantly higher (Fig. 4b), while hepatic *Cyp7a1* (Fig. 4c), BA volume (Fig. 4d,e) and total BA amount (Fig. 4f) were lower. These data demonstrate that KLF15 regulation of *Fgf15* occurs in a BA-independent manner.

## Discussion

The circadian nature of BA synthesis has been known for >50 years^19^. Given the importance of BAs in nutrient availability and metabolic homeostasis, the molecular basis for this oscillatory behaviour has been the subject of great interest. The present study identifies a novel KLF15-*Fgf15* pathway that regulates this process (Fig. 4g). Specifically, we establish KLF15 as a novel regulator of BA synthesis, show that KLF15 exacts this effect by repressing *Fgf15*, and demonstrate that the circadian regulation of *Fgf15* is independent of BA stimulation. These findings, in the context of elegant published work^9^, suggest a dual mechanism underlying *Fgf15* production: cyclical expression of ileal KLF15 in addition to the well-established pathway that involves BA-dependent activation of the nuclear receptor FXR. However, our work does not exclude the possibility that additional mechanisms may be operative by which KLF15 may affect *Fgf15* and consequently BA production. Indeed, we observed that KLF15 could repress *Fgf15* expression induced by a synthetic FXR agonist (Supplementary Fig. 4) indicating that, in principle, additional mechanisms may contribute. This is an intriguing question worthy of further study.

The KLF15-*Fgf15* pathway reported here may have important implications for the metabolism beyond the regulation of BA. FGF15 has been characterized as a ‘long-acting insulin’ with effects on glucose homeostasis following feeding^20^. Studies over the past decade have implicated KLF15 in regulation of gluconeogenesis in the context of nutrient deprivation^17^ raising the possibility that the antagonistic relationship between KLF15 and FGF15 may have important implications for glucose metabolism in a number of physiologic states. Finally, the fact that KLF15 has been shown to regulate glucose^16^, lipid^21^ and amino-acid^16^ metabolism coupled with the current insights related to nutrient availability suggest that KLF15 serves as a major node for systemic metabolic homeostasis.

## Methods

### Mice

All animal studies were carried out with permission, and in accordance with animal care guidelines from the Institutional Animal Care Use Committee at Case Western Reserve University and at collaborating facilities. Generation of systemic *Klf15*-null (*Klf15*^*−/−*^) mice has been previously described^11^. *Klf15*^*−/−*^ mice were backcrossed onto a C57BL/6J background for over 10 generations. Age-matched wild-type (*Klf15*^*+/+*^) and *Klf15*^*−/−*^ male mice on C57BL/6J background (Jackson Laboratory, Bar Harbor, ME, USA) were bred in our facility and used for circadian and other studies such as ileectomy and BDC. Mice were housed under light–dark (12 h/12 h) conditions: lights on at 06:00 (ZT0) and lights off at 18:00 (ZT12). Mice had free access to standard chow and water. Liver-specific *Klf15*-knockout mice were generated by breeding *Alb-Cre* mice and floxed-*Klf15* mice to generate *Alb-Cre*/floxed-*Klf15* (Li-KO) mice. For circadian studies, 8–12-week-old male mice were killed with isoflurane every 4 h for 24 h. Blood and tissues were harvested for mRNA, protein and other assays.

### Cell culture and reagents

Hepa1-6 (ATCC, Manassas, VA, USA) hepatocytes were maintained in DMEM (Invitrogen/Life Technologies, Grand Island, NY, USA) supplemented with 10% foetal bovine serum (FBS) and 1% penicillin/streptomycin. Murine CT26 colonic cells (ATCC) were maintained in RPMI media (Invitrogen) supplemented with 20% FBS and 1% penicillin/streptomycin. Human Caco2 colonic cells (ATCC) were maintained in EMEM (ATCC) supplemented with 20% FBS and 1% penicillin/streptomycin. Primary mouse small intestine epithelial cells were purchased from Cell Biologics Inc. (Chicago, IL, USA) and cultured in epithelial cell medium containing 0.1% epidermal growth factor, 0.1% insulin-transferrin sodium selenite, 0.1% hydrocortisone, 5% FBS, 1% L-glutamine and a 1% antibiotic-antimycotic solution on gelatin (Sigma-Aldrich, St Louis, MO, USA)-coated plates. GW4064 was purchased from Sigma-Aldrich.

### Isolation of primary mouse hepatocytes

Mice (8-week-old male) were anaesthetized by ketamine/xylazine. All media were purchased from Gibco/Life Technologies. Following dissection to expose the inferior vena cava (IVC), a catheter was inserted into the IVC, tightened by a suture and then connected to the perfusion pump with liver perfusion media. The thorax was quickly opened to clamp off the IVC above the diaphragm and the portal vein was cut. The perfusion was continued at 8 ml min^−1^ for 5 min. The liver was then perfused with collagenase I containing liver digestion media with at 8 ml min^−1^ for 6 min. Digested liver was minced and shaken vigorously to release hepatocytes in cold hepatocyte wash media. The suspension was filtered through a 70-μm cell strainer and centrifuged at 50*g* for 3 min. Hepatocytes were resuspended by William's E media, seeded in collagen I-coated plates and cultured in HepatoZYME serum-free media.

### RNA purification, reverse transcription (RT) and RT–qPCR

RNA was extracted from mouse tissues or cells using column-based Aurum total RNA fatty and fibrous tissue kit following the manufacturer’s instructions (Bio-Rad, Hercules, CA, USA). Purified RNA (1 μg) was subject to reverse transcription using iScript Reverse Transcription Supermix for RT–qPCR kit (Bio-Rad). Resulting complementary DNA (cDNA) was analysed by RT–qPCR. Briefly, 2 μl of cDNA and 1 μl of each primer were mixed with TaqMan qPCR Master Mix (Applied Biosystems/Life Technologies). Reactions were performed in 384-well format using an ABI One Plus instrument (Applied Biosystems). Relative mRNA levels were calculated using the comparative Ct method normalized to *18S*. All the primers and probes were designed using Primer Express Software (Roche, Indianapolis, IN, USA) for *Cyp7a1*, *Cyp7b1*, *Cyp27a1*, *Fxr*, *Shp*, *Klf15*, *Fgf15*, liver receptor homologue-1 (*Lrh1/Nr5a2*), hepatocyte nuclear factor 4a (*Hnf4a/Nr2a1*), liver X receptor (*Lxr/Nr1h3*), *Fgfr4* and *18S* (Supplementary Table 1).

### Molecular biology

Adenoviral overexpression studies in mouse primary small intestinal epithelial cells were performed using an adenoviral construct carrying the mouse *Klf15* gene (ad*Klf15*) or empty control virus (*Ctl*) as previously described^17^. Knockdown of *Klf15* was achieved using a *Klf15* shRNA construct (sh*Klf15*) or empty control virus shRNA (*Ctl*) as previously described^21^. To clone the *Fgf15* promoter reporter construct, mouse genomic DNA was first isolated from mouse liver using a genomic DNA isolation kit (Qiagen, Germantown, MD, USA). The mouse *Fgf15* promoter from −2,952 to −60 (2,893 bp) was amplified from genomic DNA by PCR (CloneAmp HiFi PCR Premix, Clontech, Mountain View, CA, USA) and inserted into a pGL3 luciferase reporter vector using restriction sites digested by Nhe I and Xho I (New England Biolabs Inc., Ipswich, MA, USA). The sequences of primers for *Fgf15* promoter: forward, 5′-gatcGCTAGCGCTGTCTTCAGACACAC-3′ and reverse, 5′-GCTCGGGCCACCGCACCTCGAGgatc-3′. ISH of *Fgf15* expression in the mouse ileum was carried out using a truncated clone of *Fgf15* cDNA as an ISH probe, which has been previously described^22^. RNA extraction from *Klf15*^*−/−*^ mouse ileum and RT were described above. The cDNA was subjected to PCR to amplify the *Fgf15* cDNA fragment. The sequences of primers for *Fgf15* cDNA: forward, 5′-CCAGTTGCTTTGTGGGTTGTGCCT-3′ and reverse, 5′- AAGTTCACGGGACCTTGGGGTTTTC-3′. ISH of *Klf15* expression in the mouse ileum was carried out using a truncated clone of *Klf15* cDNA as an ISH probe. RNA was extracted from *Klf15*^*+/+*^ mouse ileum and RT. The cDNA was subjected to PCR to amplify the *Klf15* cDNA fragment. The sequences of primers for *Klf15* cDNA: forward, 5′-TGTGAAGCCGTACCAGTGTC-3′ and reverse, 5′-CCTGTCAAAACAAGCTAGTTCAA-3′. The amplified cDNA fragments were inserted into the pCR2.1-TOPO vector (Invitrogen).

### Knockdown of Fgfr4 *in vivo*

Knockdown of liver *Fgfr4* was achieved using commercial *Fgfr4* shRNA construct (sh*Fgfr4*) or empty control virus (*Ctl*) provided by Vector Lab (Philadelphia, PA, USA). Mice (8–12-week-old male) were used for the tail-vein injection with shFgfr4 or control constructs (10^9^ plaque-forming unit per mouse). The mice were killed 3 days after infection for the biochemical and molecular studies.

### Western blotting assay

Liver samples were homogenized in modified RIPA buffer containing 50 mM Tris, 150 mM NaCl, 1 mM EDTA, 1% Triton X-100, 0.5% sodium deoxycholate and 1% SDS supplemented with protease and phosphatase inhibitors (Roche). CYP7A1 (Catalog #: sc25536, 200 μg ml^−1^, 1:500 dilution), CYP7B1 (Catalog #: sc26087, 200 μg ml^−1^, 1:500 dilution) and β-actin (Catalog #: sc47778, 200 μg ml^−1^, 1:1,000 dilution) antibodies were purchased from Santa Cruz and KLF15 (Catalog #: ab2647, 0.5 mg ml^−1^, 1:1,000 dilution) antibody was from Abcam (Cambridge, MA, USA) for immunoblot analyses. Immunoblot signals were quantified using ImageJ (ImageJ 1.48v, NIH). Full-length images of immunoblots are shown in Supplementary Figures 7–8.

### Tissue harvest and biochemical analysis of BAs

BA extraction was performed as previously descrived^23^,^24^. BAs in the liver, intestine and GB were extracted with 95% ethanol once overnight. Tissues were then extracted by 80% ethanol once for 2 h, and further extracted by chloroform/methanol (2:1) once for 2 h at 50 °C. BAs were determined using a bile acid assay kit (Genzyme Diagnostic, Framingham, MA, USA).

### Ileectomy or resection of ileum and jejunum

Mice (8–12-week-old male) were used for surgery. Mice were anaesthetized with isoflurane (1–2%), and placed on a temperature-controlled small animal surgical table to maintain body temperature during surgery. A midline incision was made in the abdomen to expose the intestine. The segment of ∼90% ileum or ileum and jejunum was cut-off, and warm saline and antibiotics were used to wash the cut ends. Both cut ends were further trimmed off about 2 mm, and then anastomosed with 7-0 suture. The surgery area was washed with warm saline and antibiotics. The incision of abdomen was closed with 5-0 suture. Blood and tissues were harvested for molecular and biochemical analyses 24 h after surgery.

### Bile duct catheterization (BDC)

Mice (8–12-week-old male) were used for sham or BDC. Mice were anaesthetized with isoflurane (1–2%), and placed on a temperature-controlled small animal surgical table. A midline incision in the abdomen was made to expose the bile duct. The common bile duct was catheterized at the upper margin of the pancreas using a segment of micro-miniature tubing (SUBL-190, Braintree Scientific Inc., Braintree, MA, USA) connected to a silicone tubing (SIL-025, Braintree Scientific Inc.). The incision of subcutaneous membrane was closed with 5-0 sutures. The end of the tubing was left out of the abdomen, and connected to another bile collection tubing (0.062 inch ID, Helix Medical, LLC, Carpentaria, CA, USA). The collection tubing was rolled and placed subcutaneously. The surgery area was washed with warm saline and antibiotics solution. The incision of abdomen skin was closed with 5-0 sutures. Bile was harvested from the tube twice at ZT2 and ZT14 after the BDC. After the second collection, tissues and blood were harvested for further analyses.

### ELISA assay

Mouse FGF15 ELISA kit (Catalog #: MBS161951) was purchased from MyBioSource (San Diego, CA, USA). Mouse blood was collected by heparin rinsed syringe, stored in EDTA-coated tubes and spun at 300*g* for 20 min. Plasma or FGF15 standards (40 μl per each) were added to the ELISA plate, which had been pre-coated with FGF15 monoclonal antibody. Anti-FGF15 antibody labelled with biotin was added and incubated with streptavidin-horseradish peroxidase to form immune complex. The plate was incubated for 1 h at 37 °C without exposure to the light. Nonspecific binding was washed away, and substrates A and B were added to the wells. The absorbance was read at 450 nm. The absorbance and the concentration of mouse FGF15 were positively correlated. A background of ∼30 ng l^−1^ was observed when *Fgf15*-knockout mouse plasma was used for FGF15 measurement. Total blood volume per mouse was multiplied with the FGF15 concentration to generate total circulating FGF15 amount.

### Reporter assay

Mouse *Cyp7a1-Luc* reporter construct (1 kb), and human *LXR*, *LRH1*, *HNF4A* and *RXR* expression plasmids were generously provided by Dr John Chiang at Northeast Ohio Medical University (Rootstown, OH, USA). Hepa1-6 cells were transfected with *Cyp7a1-Luc*, and *LXR*, *LRH1*, *HNF4A*, *RXR*, *Flag-Klf15* and/or *pcDNA3.1* (*Control*, *Ctl)* constructs using X-treme GENE HP DNA transfection reagent (Roche). Caco2 cells were transfected with *Fgf15-Luc* reporter constructs and *Flag-Klf15* plasmids. Luciferase activity in transfected cells was measured using a luciferase reporter assay system (Promega, Madison, WI, USA). Luciferase activity was normalized to total cell protein concentration (BCA kit, Pierce Biotechnology/Thermo Scientific, Rockford, IL, USA).

### ChIP assay

Mouse colon cell line CT26 cells were subjected to the ChIP analysis using a commercial ChIP assay kit (Millipore, Billerica, MA, USA). Briefly, cells were cultured to ∼70% confluence and then transfected with *Flag-Klf15* or *pcDNA3.1* constructs for 24 h. Cells were fixed with fresh 1% formaldehyde at room temperature for 10 min, and then neutralized by glycine. Cells were washed by PBS, and collected and pelleted at 800*g* for 5 min. Cells were lysed with cell lysis buffer to release nuclei, which were then lysed with nucleus lysis buffer. The nuclear lysates were sonicated on ice with the sonicator (Diagenode, Denville, NJ, USA) with 30% output for 6 × 10 s. The sonicated chromatin was immunoprecipitated using anti-Flag (Sigma) or non-immune IgG (Epigetek Group Inc., Farmingdale, NY, USA) and bound to Dynabeads. The yielded chromatin fragments were analysed by ChIP primers using SYBR GreenERTM PCR Master Mix (Roche). Five pairs of primers were used for ChIP of the *Fgf15* promoter and expression regions (Supplementary Table 2). The relative abundance was normalized to abundance of IgG as previously described^25^.

### *In situ* hybridization

Samples were fixed overnight with 4% paraformaldehyde and dehydrated through an ethanol series. Samples were then paraffin embedded and sectioned. Radioactive ISHs were performed using previously described protocols^26^. Probes for *Klf15* and *Fgf15* were described in Molecular Biology section above. Brightness and contrast of ISH images were adjusted identically in all control and mutant images using Adobe Photoshop.

### Lipid infusion and absorption

The method used has been described in detail by Tso and Simmonds^27^. In brief, mice were infused with a lipid diet with labelled [^3^H] triolein (TG) and [^14^C] cholesterol into the duodenum at 0.3 ml h^−1^ for 6 h. At the end of infusion, the animals were anaesthesized and the small intestine (from duodenum to ileum) was cut into four equal segments referred to as L1–L4. L1 contains luminal content from proximal segment of small intestine, duodenum; L2 and L3 contain luminal content from jejunum; L4 contains luminal content from ileum. Luminal lipids were harvested by washing each intestinal segment with 0.5% sodium taurocholate in saline and radioactivity of luminal contents measured by liquid scintillation counting. The presence of higher amounts of labelled TG or cholesterol in the duodenal lumen indicated reduced lipid absorption.

### Statistical analysis

All data were presented as mean±s.e.m. (*n* as indicated in the figure legends). The statistical difference between the two individual groups was assessed using Student’s *t*-test. For circadian mouse studies, the GraphPad software Prism (Prism 5, GraphPad Software Inc.) was used. The statistical significance between time points to assess the rhythmicity was performed using one-way analysis of variance. Circadian differences between *Klf15*^*+/+*^ and *Klf15*^*−/−*^ mice were assessed using two-way analysis of variance followed by Bonferroni post-test. Normal distribution was assumed for all experimental groups. *P*<0.05 was considered statistically significant.

## Additional information

**How to cite this article:** Han, S. *et al*. Circadian control of bile acid synthesis by a KLF15-*Fgf15* axis. *Nat. Commun.* 6:7231 doi: 10.1038/ncomms8231 (2015).

## Supplementary Material

Supplementary Information Supplementary Figures 1-8 and Supplementary Tables 1-2
