# Corrigendum: Circadian control of bile acid synthesis by a KLF15-*Fgf15* axis

**DOI:** 10.1038/ncomms9270

**Published:** 2015-08-27

**Authors:** Shuxin Han, Rongli Zhang, Rajan Jain, Hong Shi, Lilei Zhang, Guangjin Zhou, Panjamaporn Sangwung, Derin Tugal, G. Brandon Atkins, Domenick A. Prosdocimo, Yuan Lu, Xiaonan Han, Patrick Tso, Xudong Liao, Jonathan A. Epstein, Mukesh K. Jain

*Nature Communications* 6: Article number: 723110.1038/ncomms8231 (2015); Published: 06042015; Updated: 08272015.

The original version of this Article incorrectly named the author Shuxin Han as Sean Han. This has now been corrected in both the PDF and HTML versions of the Article.

