# Supplementary material for: Circadian control of bile acid synthesis by a KLF15-Fgf15 axis
Source: Nat Commun. 2015 Jun 4;6:7231. doi: 10.1038/ncomms8231 (PMC4457302; doi:10.1038/ncomms8231)
Supplement: Supplementary Information — Supplementary Figures 1-8 and Supplementary Tables 1-2 [file ncomms8231-s1.pdf]

Han et al.
Supplementary Figure 1

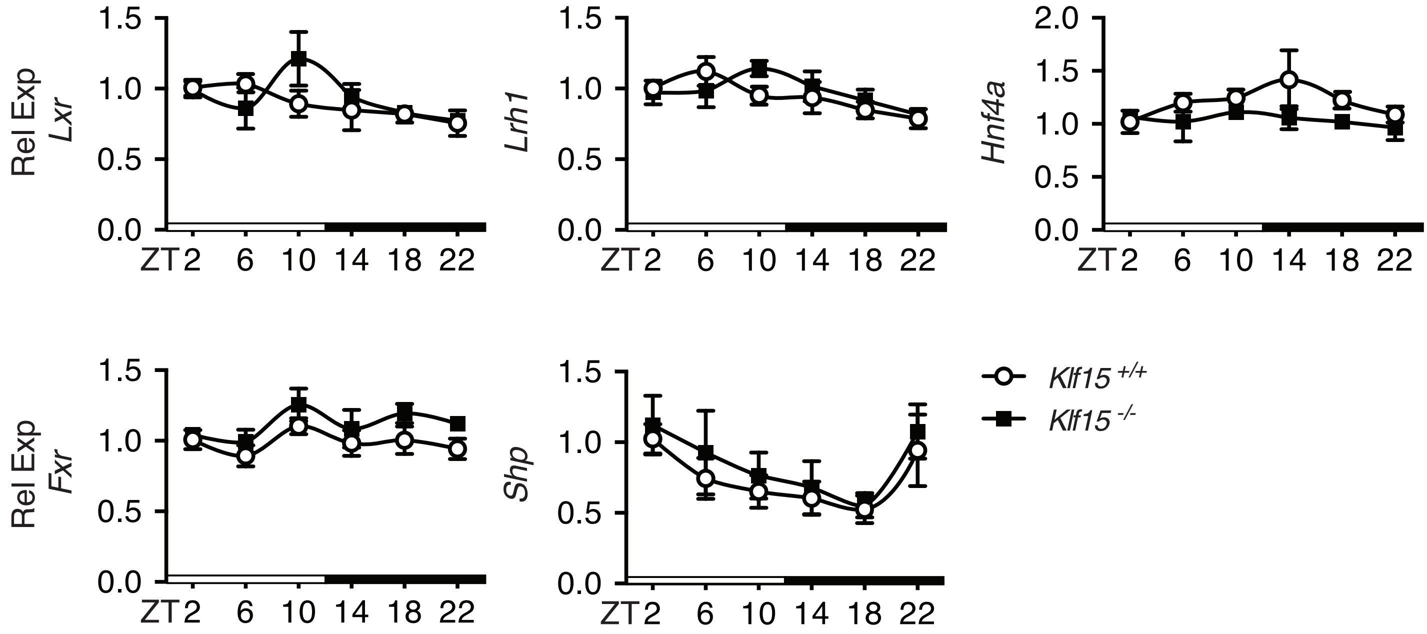

Supplementary Figure 1. Systemic *Klf15* deficiency has no effect on mRNA expression of known hepatic BA synthesis transcription factors. Quantitative RT-PCR analysis of circadian relative expression (Rel Exp) of *Lxr*, *Lrh1*, *Hnf4a*, *Fxr*, and *Shp* mRNA in *Klf15* $^{+/+}$ and *Klf15* $^{-/-}$ mouse livers (n=5). Statistical significance of circadian rhythm for each genotype was analyzed using ANOVA.

Han et al.
Supplementary Figure 2

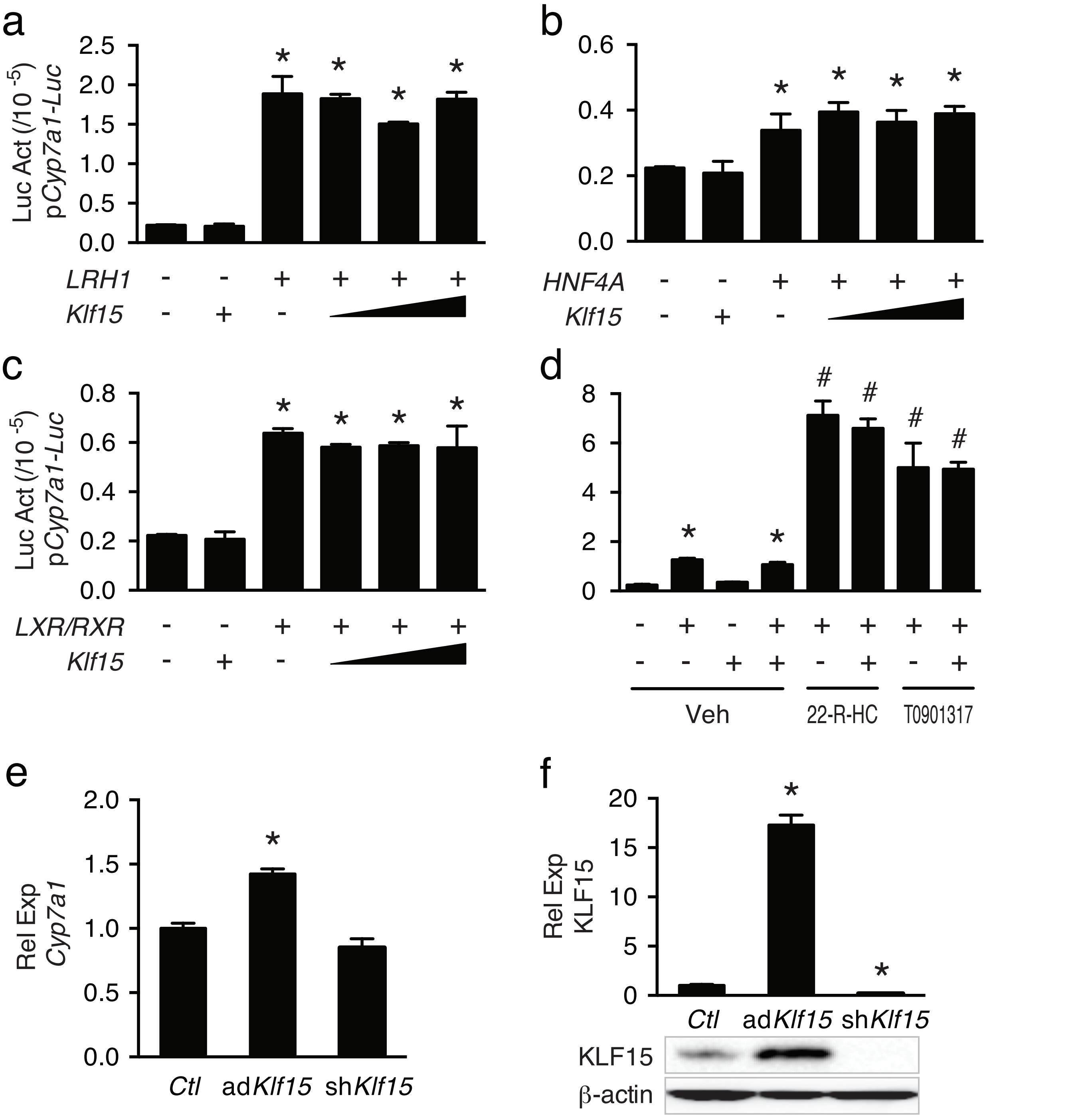

Supplementary Figure 2. Minimal effect on *Cyp7a1* reporter activity or mRNA expression in mouse hepatocytes with *Klf15* overexpression or knockdown. (a-d) Reporter assays with mouse *Cyp7a1* promoter luciferase reporter (*Cyp7a1-Luc*) in mouse hepatocyte cell line Hepa1-6. Cells were cotransfected with Klf15 and/or human *LRH1*, *HNF4A*, or *LXR/RXR* followed with treatment of vehicle (Veh), or LXR endogenous ligand 22(R)-hydroxycholesterol (22-R-HC) (2.5 μM) or specific agonist T0901317 (1.5 μM) for 16 h. (e) Effects of adenoviral overexpression and shRNA knockdown of *Klf15* on *Cyp7a1* and *Klf15* mRNA expression in primary mouse hepatocytes. The basal level of *Cyp7a1* mRNA was significantly reduced in the isolated primary mouse hepatocytes (Ct value ~31) compared with the level in the liver (Ct value ~25). (f) Effects of adenoviral overexpression and shRNA knockdown of KLF15 protein expression (representative of three experiments) and quantification. * indicates $p < 0.05$, compared to *Ctl*. # indicates $p < 0.05$, compared to Hepa1-6 cells cotransfected with *LXR/RXR*.

Han et al.
Supplementary Figure 3

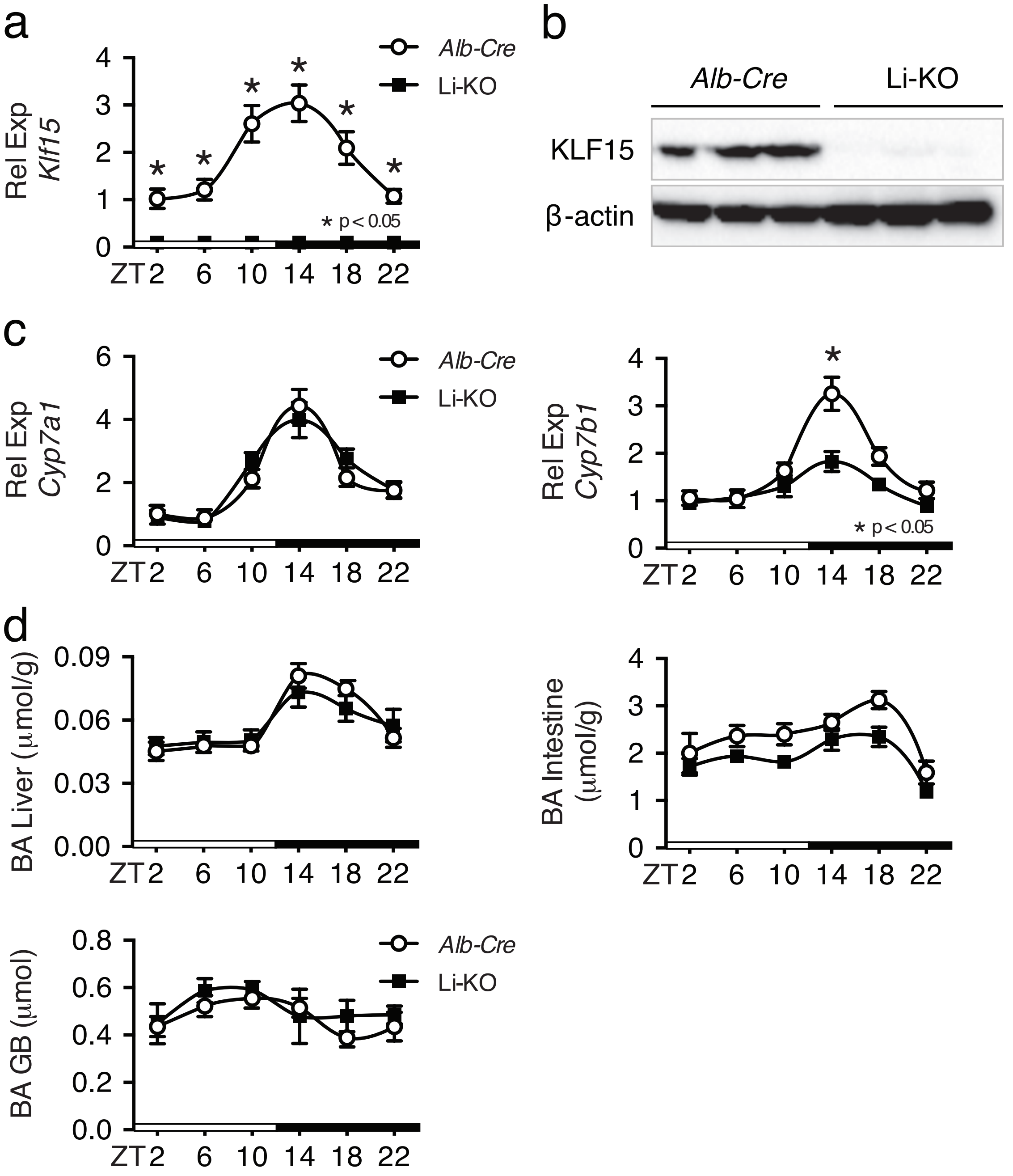

Supplementary Figure 3. Non-hepatic basis for KLF15 regulation of *Cyp7a1* expression and BA pools. (a) *Klf15* mRNA expression exhibits endogenous circadian rhythm in *Alb-Cre* mouse livers ($p < 0.01$), but the rhythm was abolished in liver-specific *Klf15* knockout (Li-KO) mouse livers (n=8 per time point). (b) Immunoblot of KLF15 in *Alb-Cre* and Li-KO mouse livers (n=3). (c) The levels of *Cyp7a1* mRNA exhibit a similar circadian pattern in *Alb-Cre* and Li-KO mouse livers. The circadian rhythm of *Cyp7b1* mRNA expression observed in *Alb-Cre* mouse livers ($p < 0.01$) was attenuated in Li-KO mouse livers ($p < 0.01$) (n=8 per time point). (d) The circadian rhythms of BA in *Alb-Cre* mouse livers, intestines, GB were maintained in Li-KO mice (n=5). Statistical significance of circadian rhythm for each genotype was analyzed using ANOVA followed by Bonferroni posttest. * indicates $p < 0.01$, compared to *Alb-Cre* mice at indicated time points.

Han et al.
Supplementary Figure 4

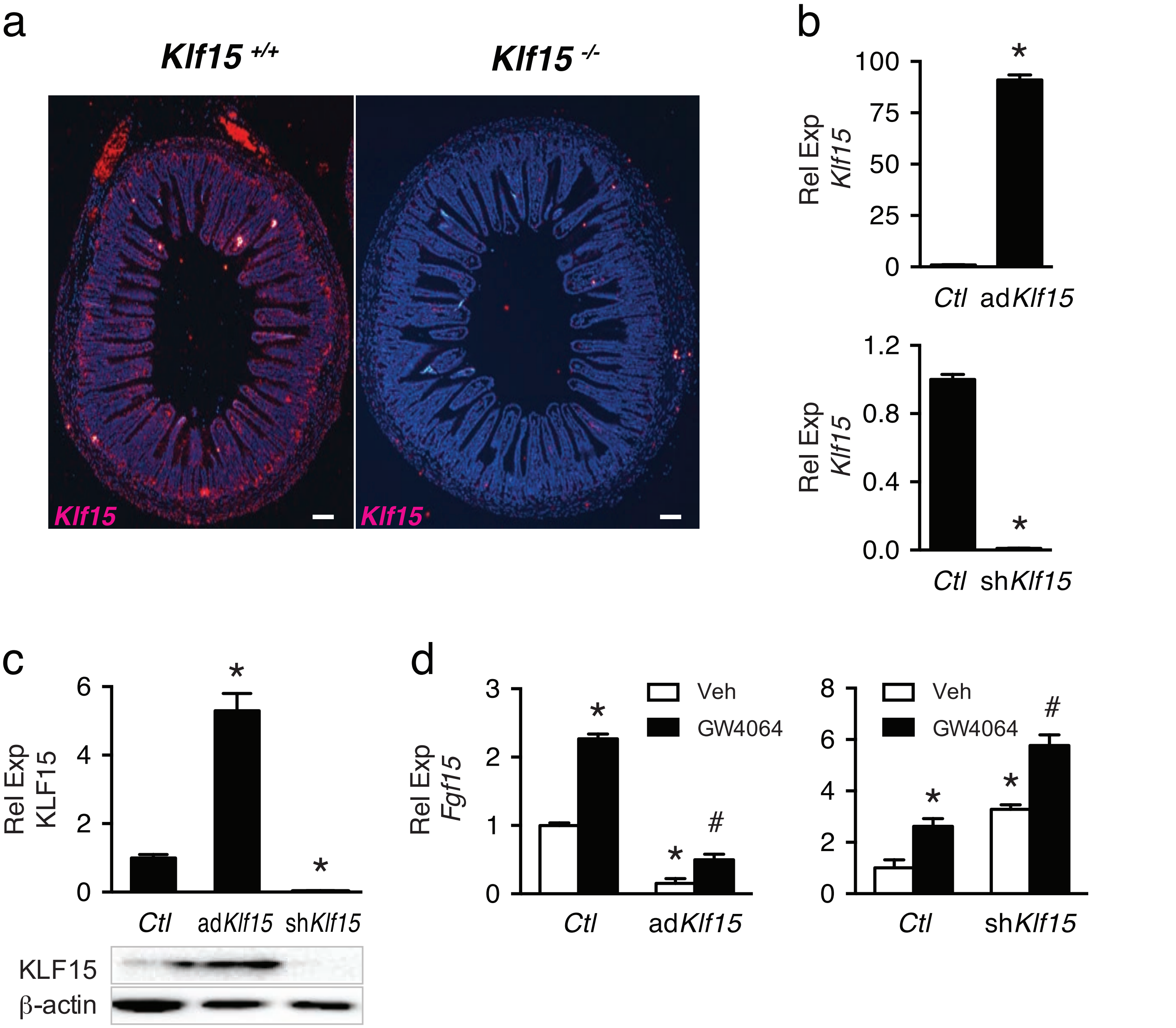

Supplementary Figure 4. KLF15 inhibition of *Fgf15* in an FXR-independent manner. (a) *In situ* hybridization analysis of *Klf15* expression in ileal epithelium. Scale bars: 100 µm. (b) QPCR analysis of adenoviral Klf15 overexpression and shRNA knockdown in primary small intestinal epithelial cells. (c) Immunblotting analysis and quantification of adenovirus-based KLF15 overexpression and knockdown in primary small intestinal epithelial cells. (d) Effects of adenoviral overexpression and shRNA knockdown of *Klf15* on *Fgf15* mRNA expression in primary mouse small intestine epithelial cells with treatment of vehicle (Veh) or GW4064 (1 µM) for 16 h. Statistical significance was assessed using Student's *t*-test. * indicates $p < 0.05$, compared with *Ctl*. # indicates $p < 0.05$, compared with Veh-treated epithelial cells infected with ad*Klf15* or sh*Klf15*.

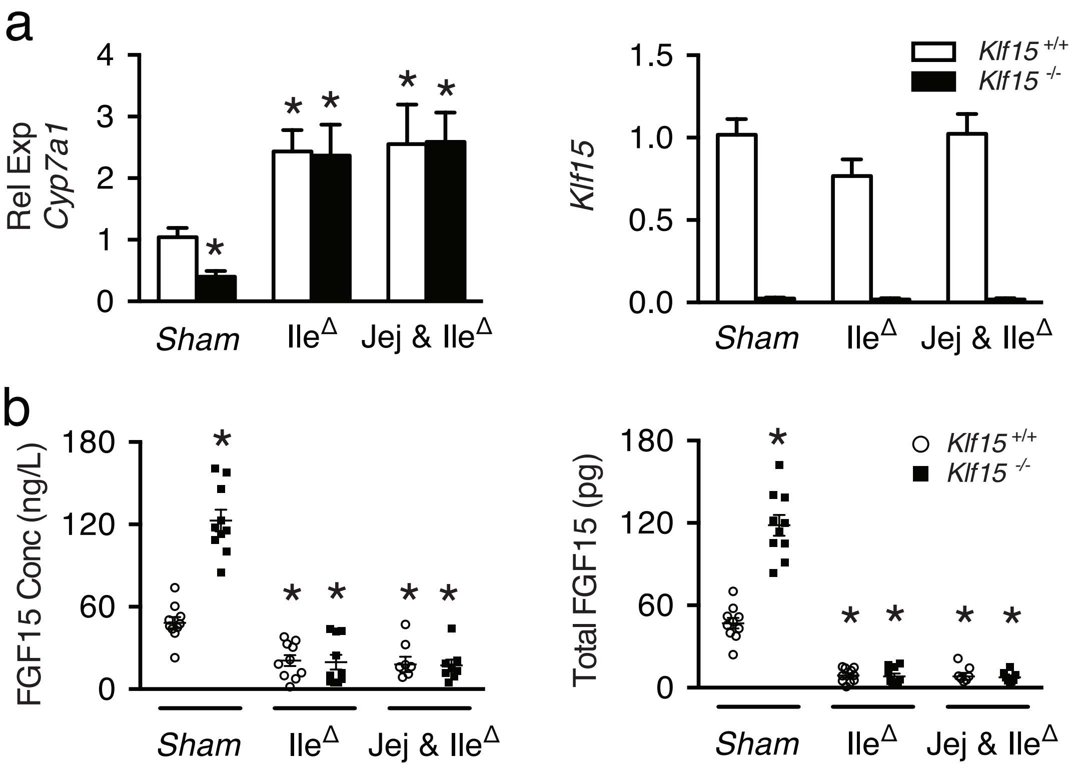

Supplementary Figure 5. Resection of both jejunum and ileum restore blood FGF15 levels, and liver *Cyp7a1* expression in *Klf15*$^{-/-}$ mice. (a) mRNA expression of hepatic *Cyp7a1* and *Klf15* in *Klf15*$^{+/+}$ and *Klf15*$^{-/-}$ mice at ZT14 after sham, Ile$^{\Delta}$, or resection of both jejunum and ileum (Jej & Ile$^{\Delta}$) surgery (n=5). (b) FGF15 protein concentration (left) and total amount (right) in blood from *Klf15*$^{+/+}$ and *Klf15*$^{-/-}$ mice at ZT14 after sham, Ile$^{\Delta}$, or Jej & Ile$^{\Delta}$ surgery (n=8). Statistical significance was assessed using Student's *t*-test. * indicates $p < 0.05$, compared to sham *Klf15*$^{+/+}$ mice.

Han et al.
Supplementary Figure 6

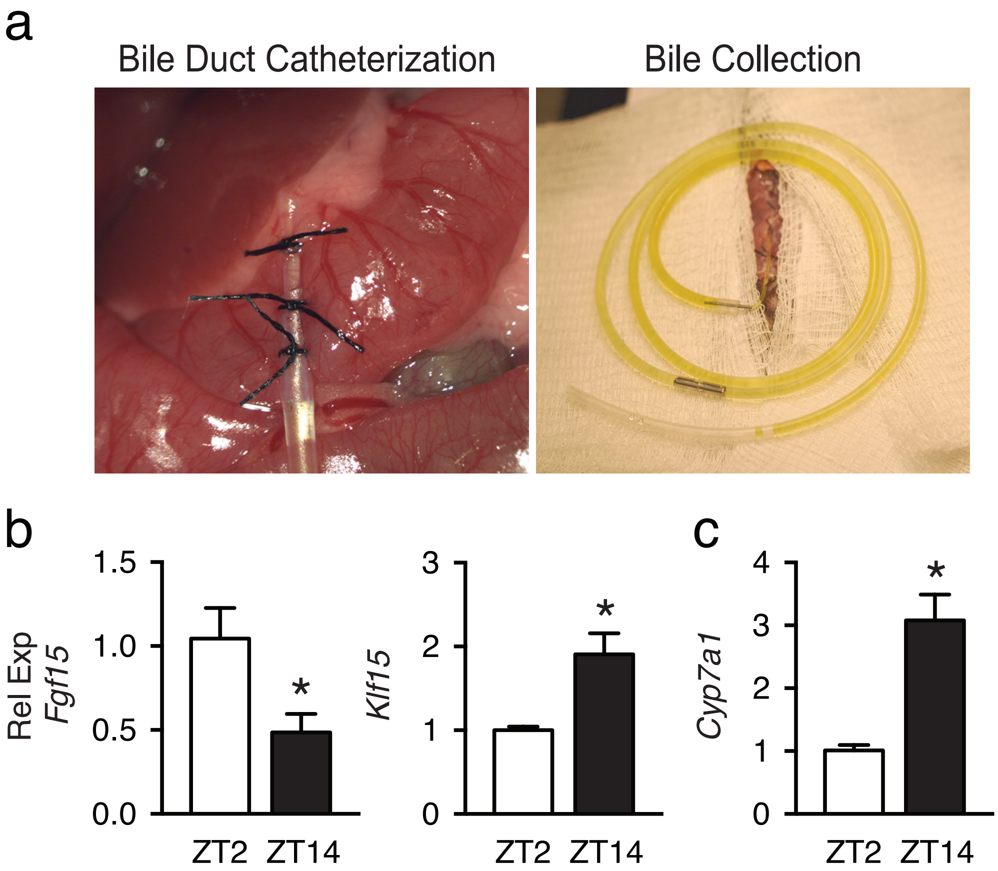

Supplementary Figure 6. Maintained oscillation of ileal *Fgf15* and *Klf15*, and hepatic *Cyp7a1* after bile duct catheterization. (a) Surgical pictures of BDC and bile captured by collection tubing. (b) Expression of ileal *Fgf15* and *Klf15* mRNA levels at ZT2 and ZT14 in BDC *Klf15* $^{+/+}$ mice. (c) Expression of liver *Cyp7a1* mRNA at ZT2 and ZT14 in BDC *Klf15* $^{+/+}$ mice (n=4). Statistical significance between two time points was assessed using Student's *t*-test. * indicates $p < 0.05$, compared to ZT2.

Han et al.
Supplementary Figure 7

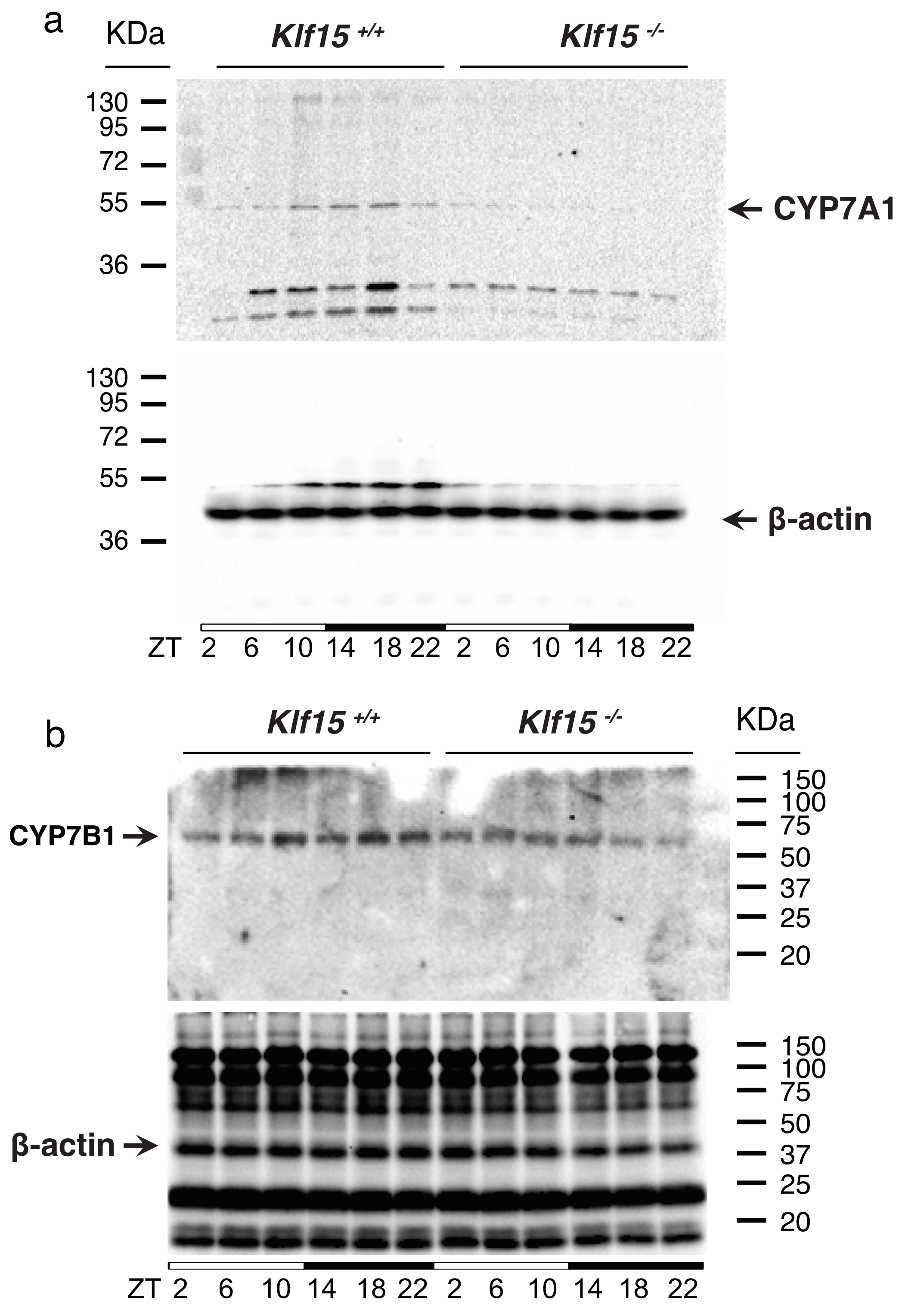

Supplementary Figure 7. Immunoblotting analysis of mouse liver CYP7A1 and CYP7B1 protein expression in a circadian fashion. (a) Immunoblot of CYP7A1 and β-actin (representative of three experiments). (b) Immunoblot of CYP7B1 and β-actin (representative of three experiments).

Han et al.
Supplementary Figure 8

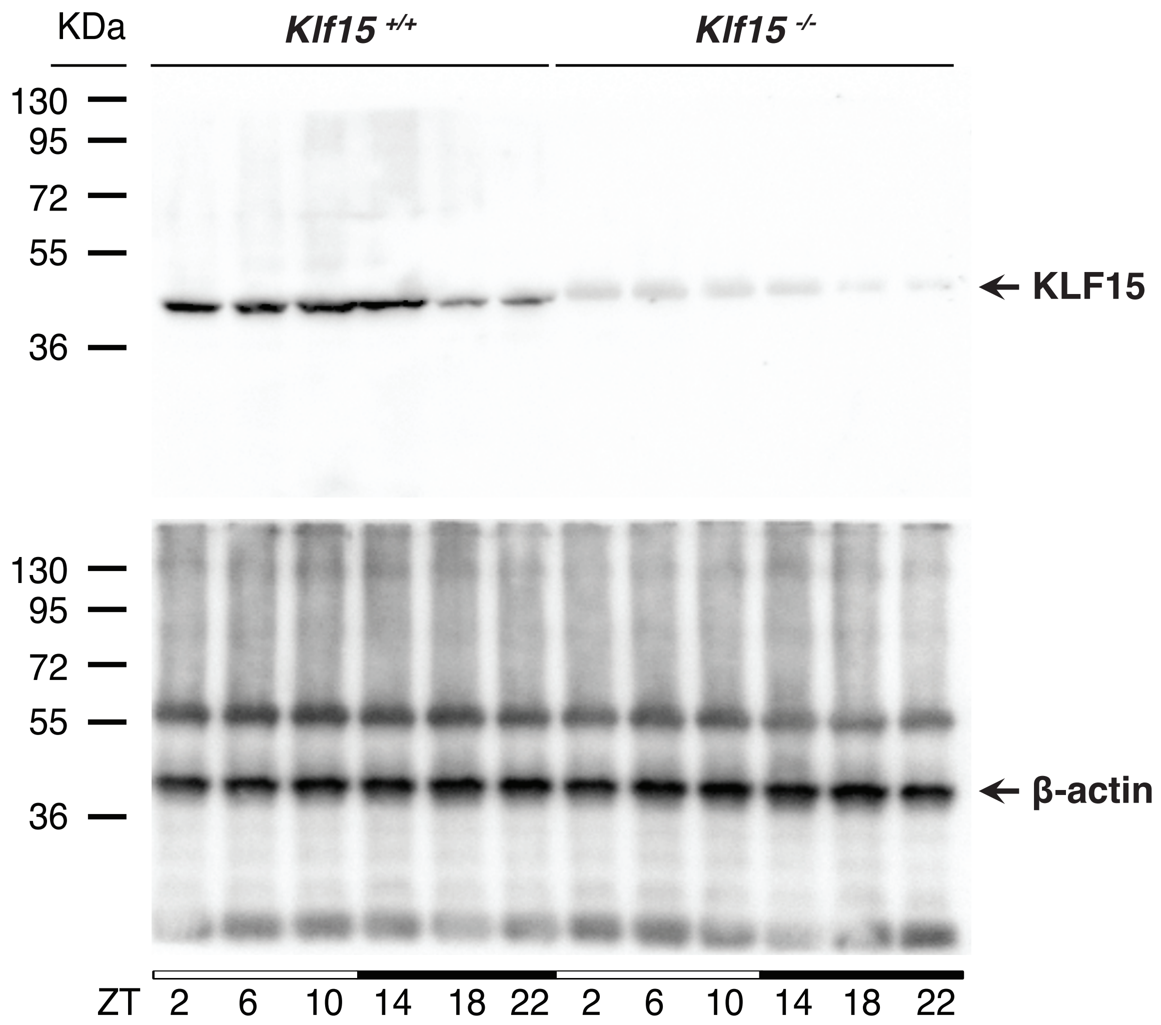

Supplementary Figure 8. Immunoblotting analysis of mouse ileal KLF15 and β-actin protein expression in a circadian fashion (representative of three experiments).

Supplementary Table 1. Primer sequences and probes used for RT-qPCR analysis.

Target	Forward primer	Reverse primer	Probe
Cyp7a1	TCAAGCAAACACCATTCCTG	GGCTGCTTTCATTGCTTCA	50
Cyp7b1	AATTGGACAGCTTGGTCTGC	TTCTCGGATGATGCTGGAGT	99
Cyp27a1	CCTCACCTATGGGATCTTCATC	TTTAAGGCATCCGTGTAGAGC	66
Fxr	GGGATGTTGGCTGAATGTATG	CACATTTTTCCTTAGCCGTTTAG	47
Shp	TTGCACCTGCATCTCACAG	AGCACACAGACGCACAGG	96
Klf15	ACAGGCGAGAAGCCCTTT	CATCTGAGCGGGAAAACCT	64
Fgf15	GGCAAGATATACGGGCTGAT	TCCATTTCCTCCCTGAAGGT	69
Lrh1	AACGATGTCCCTACTGTCGATT	CATGCGGTCGGCTCTTAC	11
Hnf4a	CAGCAATGGACAGATGTGTGA	TGGTGATGGCTGTGGAGTC	27
Lxr	TGTGCGCTCAGCTCTTGT	TGGAGCCCTGGACATTACC	71
Fgfr4	AATCGTATTGGAGGCATTCG	TCCGAGGGTACCACACTTTC	47
18S	AAACGGCTACCACATCCAAG	TACAGGGCCTCGAAAGAGTC	74

Supplementary Table 2. Primer sequences for ChIP-qPCR analysis of the *Fgf15* promoter (P) and expression (E) regions.

Fgf15 gene region	Forward primer	Reverse primer
P 1	AGTTTACCGTTCTCCCAGA	GATGCCAGAGGTGTGATGAA
P 2	GTCCCATTTTCTACCTTGTTCAGA	GGGCCGCCATAAGAACATT
P 3	CTGGTCAGTCCACCCTTCTC	CTGTCTGCA CGTCCTCTGC
E 1	TCCCTTAGGACCCAGAAGCA	CCCAGCTCCAGTCTGGAAGT
E 2	GGCTAACTGCTGAGTCCCATT	AAGCCAGGAGAGGAGGCTTT
